# Nitric oxide inhibition strategies

**DOI:** 10.4155/fso.15.35

**Published:** 2015-08-01

**Authors:** Vivian (Wai Chong) Wong, Ethan Lerner

**Affiliations:** 1 Department of Dermatology, Rhode Island Hospital/Brown University, 593 Eddy Street, Providence, RI 0290, USA; 2 Department of Dermatology, Cutaneous Biology Research Center, Massachusetts General Hospital & Harvard Medical School, Boston, MA 02115, USA

**Keywords:** apoptosis, programmed cell death, toxic epidermal necrolysis

## Abstract

Nitric oxide is involved in many physiologic processes. There are efforts, described elsewhere in this volume, to deliver nitric oxide to tissues as a therapy. Nitric oxide also contributes to pathophysiologic processes. Inhibiting nitric oxide or its production can thus also be of therapeutic benefit. This article addresses such inhibitory strategies.

**Figure 1.**
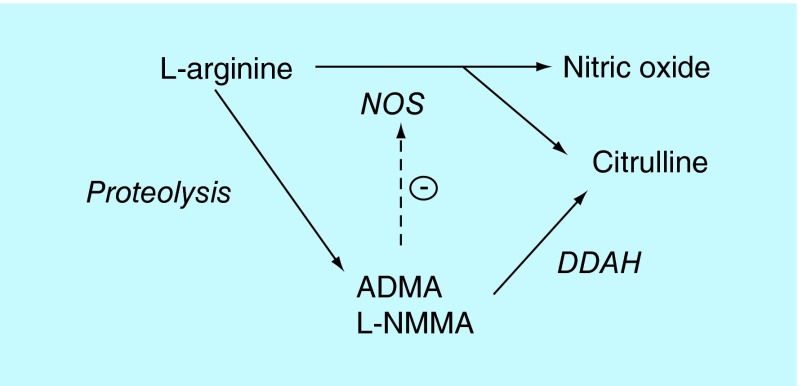
**Synthetic and degradation pathways of nitric.** Nitric oxide is synthesized, along with citrulline, from L-arginine by nitric oxide synthase. L-arginine may be proteolyzed to form methylarginines (ADMA and L-NMMA), which in turn inhibit NOS activity by competing with arginine at the active site. Methylarginines are metabolized by dimethyl-arginine-dimethyl-aminohydrolase (DDAH) into citrulline and dimethylarginine. Citrulline can be converted back to arginine by enzymes of the urea cycle [25]. NOS: Nitric oxide synthase; DDAH: dimethyl-arginine-dimethyl-aminohydrolase.

## Introduction: why would we want to increase or decrease nitric oxide?

Nitric oxide is an important biological mediator of functional and metabolic processes in nearly every organ system. This gaseous free radical moderates endothelial function, neurotransmission, immunity and cell death by activating guanylate cyclase (GC), which increases intracellular cGMP levels and cGMP-dependent protein kinase [1]. Elevation of nitric oxide and cGMP has been implicated in many pathophysiological processes. As our understanding of this powerful signaling molecule continues to grow, many are hopeful that tissue and/or site-specific inhibition of nitric oxide will have a tremendous impact in multiple medical disciplines.

## Conditions that may be due to excess nitric oxide & why

### Shock

Excessive nitric oxide production is recognized in septic shock and cardiogenic shock. Nitric oxide inhibitors revert hypotension in shock induced by cytokines, heat and hemorrhage in animal studies [2,3]. It is not surprising that nitric oxide inhibitors were once thought to be promising agents for these conditions. Methylene blue, a nitric oxide inhibitor, has been used for many years in septic shock and anaphylactic shock. It was alleged to improve mortality by elevating blood pressure and systemic vascular resistance in these clinical scenarios. Nevertheless, outcome studies on the use of nitric oxide inhibitors in cardiogenic shock delivered disappointing results. Human studies were conducted only with nonselective l-arginine analogs, which fail to reduce mortality in septic shock [4,5]. In fact, nonselective nitric oxide synthase (NOS) inhibitor treatment alone elevates mortality [6]. Additional studies are under way to further examine the role of selective NOS inhibitors in shock.

### Dermatology

Nitric oxide is present in various types of skin cells, including keratinocytes, melanocytes, Langerhans cells, fibroblasts and endothelial cells. Increased nitric oxide production is demonstrated in psoriasis, atopic dermatitis, irritant dermatitis, allergic dermatitis, lupus erythematous, sunburn-induced flushing, nerve-mediated flushing and skin swelling [7,8]. Given the role of nitric oxide as an endothelium-derived vasodilator, nitric oxide could be a culprit for causing the redness and inflammation of rosacea [8]. NOS inhibitors are also being considered for the aforementioned dermatological conditions.

Nitric oxide may also contribute to epidermal apoptosis and necrosis, a phenomenon observed in blistering drug reactions. In fact, inducible nitric oxide is expressed in skin biopsies from patients with toxic epidermal necrolysis and Stevens–Johnson syndrome, suggesting that NOS inhibitors are potential therapeutic targets for these challenging conditions [9].

Previously unknown roles for nitric oxide in dermatology continue to be uncovered. Nitric oxide disrupts hematopoiesis during acute graft-versus-host disease, hence facilitating secondary bacterial infection in mice [10]. Nitric oxide may also be pivotal during skin cancer therapy, by way of mediating treatment resistance to cisplatin treatment in melanoma cells *in vitro* [11].

### Neurology

Nitric oxide inhibitors generate favorable results as a migraine remedies. Nitric oxide regulates cerebral blood flow and nociception in animal models of migraine. Selective neuronal NOS (nNOS) and inducible NOS (iNOS) inhibitors are in early clinical development for treating headache and migraine [12]. Nitric oxide possibly contributes to intracerebral hemorrhage by precipitating endothelial dysfunction and secondary injury [13]. Nitric oxide is also implicated in Parkinson's disease, as its downstream massager GC is upregulated in mice models. The role of nitric oxide in epilepsy is more complicated, as evidence suggests that it may be a neuromodulator with both proconvulsive or anticonvulsive actions in animals [14].

### Oncology

Endogenous nitric oxide promotes tumor progression and metastasis through stimulation of tumor cell migration and angiogenesis *in vitro* [15]. In contrast, nitric oxide is also implicated in cellular apoptosis and necrosis *in vitro* [16]. Using NOS inhibitors in cancer patients may be a double edged sword; on the one hand, iNOS is overexpressed in tumor cells [17]; on the other hand, nitric oxide may be involved in chemosensitization [18].

## Potential side-effects of inhibiting nitric oxide

Nitric oxide inhibition could be detrimental to patients with cardiovascular and renal diseases. Nitric oxide is cardio-protective during ischemic events by causing coronary vasodilation and improving oxygen delivery. Nitric oxide inhibition also suppresses statin-induced oxygen delivery to myocardium [19]. Nitric oxide inhibition could contribute to endothelial dysfunction and inflammatory syndrome in patients with autoimmune disease, leading to an escalation of cardiovascular morbidity and mortality [20].

In patients with chronic kidney disease, nitric oxide inhibition aggravates endothelial dysfunction, vasoconstriction, blood pressure elevation and atherosclerosis, thereby worsening kidney disease progression, particularly in the setting of diabetic nephropathy [21,22]. Nitric oxide inhibition is also demonstrated in insulin resistance [23]. Erectile dysfunction and micturition disorders are also mediated by nitric oxide [24], and could be adversely affected by nitric oxide inhibition.

## Potential approaches to inhibiting nitric oxide

Targeted approaches to intervene the nitric oxide synthetic or signaling pathway are not available for clinical use. At present, potential pharmacological inhibition of nitric oxide is achieved via inhibition of NOS, inhibition of downstream mediators and nitric oxide inhibition/scavenging. Nonpharmacological ways to inhibit nitric oxide, such as gene therapy, are beyond the scope of this review.

### Inhibition of NOS

Nitric oxide synthases are enzymes that generate nitric oxide in tissues. There are three isoforms of NOS. eNOS (endothelial NOS) and nNOS (neuronal NOS) are constitutively expressed and regulated by transcription and post-transcription processes. iNOS (inducible NOS) is released *de novo* in response to inflammation. NOS inhibitors of varying degrees of potency and selectivity are available and utilized in research studies.

There are two endogenous NOS inhibitors (Figure 1). ADMA is a potent, noncompetitive NOS inhibitor, while its congener L-NMMA is a less potent, competitive NOS inhibitor. While ADMA has been shown to contribute to the inflammatory syndrome and endothelial dysfunction seen in shock, its clinical application awaits further investigation.

L-NMMA (‘Tilarginine’) is a nonselective NOS inhibitor. L-NMMA dose-dependently increases blood pressure by causing arterial vasoconstriction in humans [26]. This agent was investigated in the TRIUMPH (Tilarginine Acetate Injection in a Randomized International Study in Unstable MI Patients with Cardiogenic Shock) study with patients in North America and Europe. The study was terminated early due to a lack of clinical benefit [27]. In another randomized control trial on 12 patients with severe sepsis and hypotension, L-NMMA caused a fall in cardiac output, worsening tissue perfusion [4]. L-NMMA remains a prospective candidate for other diseases. L-NMMA may help prevent skin cancer, for its use improves sunscreen protection from sunburn, immunosuppression and photocarcinogenesis in mice [28]. L-NMMA also treats migraine attacks without aura, chronic tension-type headache and cluster headache [12].

Synthetic NOS inhibitors have been evaluated for clinical use. N(G)-methyl-l-arginine hydrochloride (546C88) is a nonselective NOS inhibitor shown to restore the balance of vasomotor tone in patients with septic shock, reducing the concomitant requirement for norepinephrine treatment. It was studied in a Phase III clinical trial in Europe, North America, South America, South Africa and Australasia. This study was terminated early because of increased mortality in this condition [29]. N-nitro-l-arginine methyl ester (L-NAME) and Ng-nitro-l-arginine (L-NArg) are other synthetic nonselective NOS inhibitors, with implications for substance abuse, since they attenuate signs of opioid withdrawal in rats [30]. L-NAME also seems promising for treating septic shock by maintaining blood pressure [31]. Chronic L-NAME treatment reduces angiogenesis in migration and invasiveness *in vitro*, pointing to its possible future use as tumor-suppressing agent [15]. 7-nitroindazole (7-NI) is a specific inhibitor of nNOS, and has anticonvulsive properties in seizure models in rodents. It has at times been shown to be proconvulsant in kainite-, nicotine- and soman-induced convulsions in rodents [14]. Aminoguanidine is a selective iNOS inhibitor that attenuates graft-versus-host disease by decreasing hematopoietic indices and concomitant susceptibility to bacterial infection in mice [10].

### Inhibition of downstream mediators

Methylene blue is an inhibitor of the soluble guanylate cyclase (sGC). It is a dye that may be safely used in humans for septic shock. It appears to provide effective protection in TNF-induced shock [32], as well as anaphylactic hypotension unresponsive to other interventions [33]. Methylene blue may also be beneficial for refractory cases of vasoplegia, a common complication of cardiopulmonary bypass, by alleviating inflammation-mediated dysregulation of endothelial function [34].

1H-[1,2,4] oxadiazolo-[4,3-a]quinoxalin-1-one (ODQ) is a selective sGC inhibitor in rats, shown to restore basal ganglia function and improve motor symptoms in Parkinson's disease [35].

### NO inhibition/scavenging

All hemoglobins are potential scavengers of NO. The chemically modified human-derived, hemoglobin conjugate, pyridoxalated hemoglobin polyoxyethylene such a scavenger. It has the potential to increase systemic blood pressure while reducing vasopressor and ventilation needs in shock. A Phase III clinical trial on its use in septic shock with systemic inflammatory response syndrome is pending publication [36].

Several herbal agents have demonstrated possible use as nitric oxide inhibitors. Notably, (-)-epigallocatechin-3-gallate (EGCG) inhibits migration of mammary cancer cell, while grape seed proanthocyanidins inhibits migration of nonsmall cell lung cancer cell, by inhibiting NOS and GC [37].

## Approaches under development

Arginase has emerged as another regulator of nitric oxide homeostasis. Arginase is an enzyme in the urea cycle that hydrolyzes L-arginine to urea and L-ornithine. It suppresses nitric oxide production through numerous mechanisms. It may contribute to endothelial dysfunction in hypertension, aging, ischemia-reperfusion, psoriasis, erectile dysfunction, arthritis, diabetes and pathological wound healing [38,39].

DDAH inhibition may provide an alternative means to inhibit nitric oxide [25]. Homocystein inhibits DDAH activity, which could explain the known effect of homocysteine to impair nitric oxide-dependent vasodilatation [40].

Nitric oxide also activates potassium, big potassium (BK) and calcium channels, most of which can be blocked pharmacologically by specific agents. It might be predicted that signaling molecules and enzyme cofactors in nitric oxide synthesis may serve as promising targets. For instance, nitric oxide activity is regulated by tetrahydrobiopterin and calmodulin. The synthesis of nitric oxide is calcium-dependent, therefore calcium mobilizing agonists could provide therapeutic targets in the future.

## Conclusion & future perspective

Nitric oxide is involved in a wide spectrum of human physiology and pathophysiology. Pharmacological agents to inhibit nitric oxide may modify or arrest the course of some of these NO-mediated pathologies. There are numerous limitations to our current knowledge on nitric oxide inhibition. We must keep in mind that most of the pharmacotherapy studies on nitric oxide inhibition were performed in animals or on small groups of patients, with a short course of treatment. The use of nitric oxide inhibitors in patients with poly-pharmacy remains a challenge, as there is inadequate understanding of interactions between nitric oxide inhibitors and other drugs. Known interactions have already been reported with statins, fibrates, thiazolidinediones, metformin, antioxidant vitamins, aspirin, n-3 polyunsaturated fatty acids and plant flavonoids. Nitric oxide provokes many cellular responses and modulates physiological functions differently depending on the organ system. Systemic nitric oxide inhibition may be limited by the widespread involvement of nitric oxide in most body systems. To further complicate matters, depending on the disease studied, changes in nitric oxide may either ameliorate or exacerbate the pathophysiology of the disease. This proves to be a particular challenge in patients with co-morbidities. Finally, there is a paucity of studies that survey the chronic use of NOS inhibitors. One animal study shows that chronic NOS inhibition may also produce long-term biological effects by enhancing early atherogenesis in animals [41]. The potential for either acute or chronic inhibition of nitric oxide has yet to reach its potential.

**Executive summary**

Elevation of nitric oxide has been implicated in many pathophysiological processes.
Nitric oxide inhibitors are promising agents for treating various cardiovascular, dermatological, neurological and oncological conditions.
At present, potential pharmacological inhibition of nitric oxide is achieved via inhibition of nitric oxide synthase, inhibition of downstream mediators and nitric oxide inhibition/scavenging.
Nitric oxide inhibition should be performed with caution in patients with renal or cardiovascular disorders.
New approaches to inhibit nitric oxide are being studied.
Our understanding of nitric oxide inhibition would benefit from large scale, long-term animal and human studies.
